# ELIXIR: providing a sustainable infrastructure for life science data at European scale

**DOI:** 10.1093/bioinformatics/btab481

**Published:** 2021-06-27

**Authors:** Jennifer Harrow, Rachel Drysdale, Andrew Smith, Susanna Repo, Jerry Lanfear, Niklas Blomberg

**Affiliations:** ELIXIR Hub, South Building, Wellcome Genome Campus, Hinxton, Cambridge CB10 1SD, UK; ELIXIR Hub, South Building, Wellcome Genome Campus, Hinxton, Cambridge CB10 1SD, UK; ELIXIR Hub, South Building, Wellcome Genome Campus, Hinxton, Cambridge CB10 1SD, UK; ELIXIR Hub, South Building, Wellcome Genome Campus, Hinxton, Cambridge CB10 1SD, UK; ELIXIR Hub, South Building, Wellcome Genome Campus, Hinxton, Cambridge CB10 1SD, UK; ELIXIR Hub, South Building, Wellcome Genome Campus, Hinxton, Cambridge CB10 1SD, UK; ELIXIR Hub, South Building, Wellcome Genome Campus, Hinxton, Cambridge CB10 1SD, UK

In 2012, the Digital Universe Study reported that ‘less than 1% of the World’s data is analyzed and less than 20% is protected’ and recommended that management plans were needed to harness the potential within these data streams (Gantz and Reinsel, 2012). Life science is a data science that is dependent on the generation, sharing and integrative analysis of vast quantities of digital data. However, these data are complex and fragmented, creating a significant barrier to their integration and reuse. Data are generated for different research purposes at thousands of facilities across the world, with diverse formats, annotations, methodologies and metadata standards. Interpreting this data is difficult and often involves integrating very large datasets from multiple sources. Certain data types such as sensitive human data archived under controlled access require protection, so data federation and sharing are a challenge in this context (Saunders *et al.*, 2019).

Since its official launch in December 2013, ELIXIR (https://elixir-europe.org/), the European infrastructure for life science data, has worked to address these challenges by bringing Europe’s national centers and core bioinformatics resources into a single, coordinated infrastructure. Many European countries have long-established national bioinformatics infrastructures that provide tools, data resources and cloud services to national and international users. ELIXIR invests in and aims to sustain these vital resources and ensure a level of interoperability that facilitates scientific discovery. Indeed, for research to thrive in a world of data abundance, all components (research data, analysis tools, standards as well as computational resources and training materials) must be FAIR—findable, accessible, interoperable and reusable (Wilkinson *et al.*, 2016). ELIXIR supports European life scientists in making data FAIR, ensuring that the benefits extend far beyond those actively participating in ELIXIR, and, additionally, encourages all funders to adopt Open Science mandates.

Here, we describe how ELIXIR has evolved to be an established research infrastructure, with a review of significant milestones achieved, and a look into the future. By linking national networks that span most of Europe’s major research centers ELIXIR is in a unique position to drive the transformation of life science’s distributed data resources within Europe to be sustainable, federated, standards-based and cost-effective.

## Building a stable and sustainable infrastructure for biological information across Europe

**Fig. 1.**
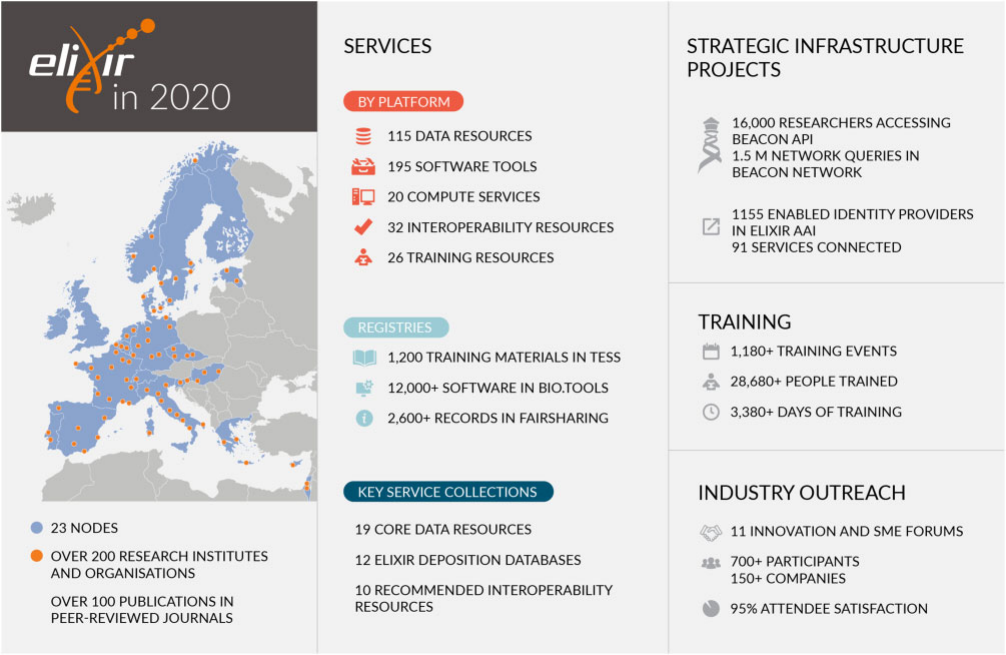
Overview of the ELIXIR infrastructure including members, services, infrastructure, training and industry events

ELIXIR is now a mature intergovernmental organization that has grown from 6 members in 2014 to 22 members and one observer in 2020. ELIXIR links more than 220 institutes and over 700 national experts dedicated to the development and operation of national services that contribute to data access, integration, training and analysis for the research community (see Fig. 1).

Fundamentally, ELIXIR is connecting national bioinformatic networks, known within ELIXIR as Nodes (https://elixir-europe.org/about-us/who-we-are/nodes). Some ELIXIR Nodes predate ELIXIR, such as EMBL-EBI and the Swiss Institute of Bioinformatics. In many other countries, ELIXIR has contributed to the formation of new national bioinformatics infrastructures, e.g. de.NBI (https://www.denbi.de; ELIXIR Germany) and ELIXIR Czech Republic (https://www.elixir-czech.cz/), both launched in 2015. The participating countries contribute membership fees, proportional to the countries Net National Income (NNI), provide ELIXIR with a technical budget that is used to fund development of shared services that support and link the nationally funded bioinformatics resources.

The Nodes guide the direction of ELIXIR in alignment with their own national priorities, as has been described, e.g. by ELIXIR Netherlands (Eijssen *et al.*, 2015), ELIXIR Switzerland (Baillie Gerritsen *et al.*, 2019) and ELIXIR Norway (Tekle *et al.*, 2018). ELIXIR aims to ensure that all life science projects, irrespective of their membership status with respect to ELIXIR, have access to advanced, scalable and long-term sustainable infrastructure across Europe. In this expanding interdisciplinary field, training of new researchers and infrastructure providers is key for success and ELIXIR runs a vibrant training network. For example, between September 2015 and March 2019, ELIXIR was able to register over 1200 training materials and train more than 19 000 people. Capacity building of this magnitude depends upon the coordinated reuse of skills, resources and materials.

Partnerships between industry and academia are increasingly important if the societal impact of scientific research is to be fully realized. For instance, access to open data and software has been shown to be of long-term value to the bioeconomy (Bousfield *et al.*, 2016; Rothe *et al.*, 2019). Industrial users of ELIXIR’s services range from large multinationals to micro-Small and Medium Enterprises (SMEs), and include the pharmaceutical, healthcare, food, agriculture and blue biotech sectors. ELIXIR has shown that many SMEs extensively rely on life science data from public resources (Garcia *et al.*, 2018). ELIXIR aims to build new partnerships and strengthen existing collaborations (Lauer et al., 2019) through its innovation and SME Forum, which has delivered 10 events over the first program, covering topics as diverse as Genomics and Health and Data-driven innovation in the agri-food industries.

## Engagement through Platforms and Communities

ELIXIR facilitates collaboration between its member institutes and researchers with two intersecting organizational groupings, the Platforms (https://elixir-europe.org/platforms) and the Communities (https://elixir-europe.org/communities). The ELIXIR Platforms (Data, Compute, Interoperability, Tools and Training) are supported by Technical Coordinators located at the ELIXIR Headquarters, with vision and strategy led by senior scientists in the Nodes. As part of joining ELIXIR, each Node contributes a set of services (https://elixir-europe.org/services) that are aligned with the Platforms, e.g. Human Protein Atlas (Uhlén *et al.*, 2015) (Data Platform—ELIXIR Sweden), CSC Cloud (https://research.csc.fi/) (Compute Platform—ELIXIR Finland), Identifiers.org (Juty et al., 2012) (Interoperability Platform—EMBL-EBI), CAMEO (Haas *et al.*, 2018) (Tools Platform—ELIXIR Switzerland) and TeSS Training Portal (Beard *et al.*, 2020) (Training Platform—ELIXIR UK). The Node-contributed services form the basis for ELIXIR development work.

**Fig. 2.**
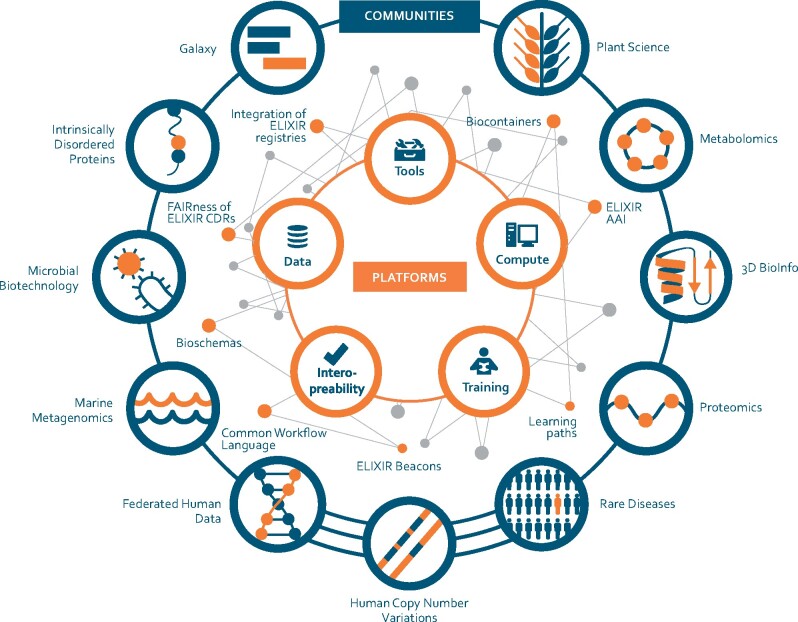
ELIXIR’s life science research Communities and Platforms are linked by Implementation Studies

Established and driven by researchers and bioinformaticians in the Nodes, the ELIXIR Communities identify the needs of domain- or technology-specific research around a specific theme, e.g. Metabolomics, Microbial Biotechnology. ELIXIR now has a total of eleven Communities (see Fig. 2) which is a significant progression from the four use cases (Federated Human Data, Marine Metagenomics, Plant Sciences and Rare Disease) that were supported by the initial Horizon 2020 ELIXIR-EXCELERATE grant. Harrow *et al.* (2021) describe how ELIXIR can support the research communities in their effective use of life science data, as exemplified by the EXCELERATE use cases.

To ensure engagement between Nodes, Platforms and Communities, ELIXIR conducts Implementation Studies (https://elixir-europe.org/about-us/implementation-studies) that develop services and bring benefit to key user communities (see e.g. Fig. 2). To date, more than 50 Implementation Studies, generally lasting between 6 months and 2 years, have been initiated. These are deliberately of short duration, timely, focused on bottlenecks highlighted by a Community or Platform, and their completion generally provides analysis components, workflows or validations. They are selected based on high scientific or technical merit and broad applicability, typically addressing different stages of the data life cycle, e.g. secure data deposition, distributed data annotation or advanced data reuse. They drive development of the service portfolio and generate opportunities for building bundles of services bespoke for particular communities.

## ELIXIR enables progress within life sciences

ELIXIR Communities and Platforms illustrate how ELIXIR fosters long-term collaborations that connect national resources into transnational infrastructure. The federated data infrastructure is held together by strong community standards, with users and data providers agreeing on conventions for annotating, depositing, finding and accessing data. ELIXIR’s distributed organization with a scientific leadership that represents 23 national bioinformatics Nodes is a powerful vehicle to consult and build consensus on joint standards and drive the implementation in national organizations and communities.

For instance, the ELIXIR Plant Sciences Community has built a common technical infrastructure and associated social practices to support plant genotype-phenotype analysis with the goal to make plant genotypic and phenotypic data easier to find, integrate and analyze, by making them FAIR (Pommier *et al.*, 2019). The Community developed and proposed an extension of the Minimal Information about Plant Phenotyping Experiments (MIAPPE) v1.0 specification (Krajewski *et al.*, 2015) and is recognized as a significant player in the global plant community and is working on MIAPPE with EMPHASIS (https://emphasis.plant-phenotyping.eu/), the ESFRI for European Plant Phenotyping, and the CGIAR (https://www.cgiar.org/), the world’s largest global agricultural research organization.

It is particularly beneficial in rapidly developing fields to bring together leading experts across national centers to collaboratively develop infrastructure and standards. ELIXIR’s Marine Metagenomics Community has addressed a lack of accessible databases by providing curated reference data that, together with benchmarking datasets and standardization of computational pipelines, provides the necessary infrastructure to enhance both academic and industrial research and development. New databases such as MarRef, MarDB, MarCat and METdb provide important new reference data sources for interpretation and species assignment within the wider marine metagenomics community (Robertsen *et al.*, 2017).

A software ‘container’ packages code with all its dependencies, enabling the application to run quickly and reliably across a range of computing environments, facilitating reproducible research. The BioContainers Implementation Study is an example of an initiative driven by the ELIXIR Tools Platform to provide a stable infrastructure for unifying software containerized solutions within ELIXIR. This infrastructure provides an access point for end-users to find, generate, store, monitor and even benchmark software containers. This has resulted in the development of a registry for containers, BioContainers (da Veiga Leprevost *et al.*, 2017), which allows researchers open access to 7.9 K containerized tools.

The Bioschemas convention, set up in 2015 to improve FAIRness of data, is driven by the ELIXIR Interoperability Platform as an open international community initiative. Bioschemas improves the findability of data in the life science resources by extending a Schema.org markup to specific biological data types. This markup can be introduced into websites, without the need for specific technical expertise, such that they become readily indexable by search engines and other services (Profiti et al., 2018). ELIXIR continues to encourage the development of Bioschemas through its yearly Biohackathon (https://www.biohackathon-europe.org/), which brings developers together from different Platforms and Communities to collaborate on preselected projects.

ELIXIR also funds strategic infrastructure projects such as AAI, the Authorization and Authentication Infrastructure (https://elixir-europe.org/services/compute/aai). AAI facilitates data access that requires specific authorization on account of its sensitive nature, e.g. human genomic data. Before data access authorization and delivery can be carried out, a researcher needs to be identified and their identity authenticated to a sufficient level of assurance. AAI provides this function. ELIXIR AAI is still in development, but already has 603 enabled identity providers and 2470 registered users organized in 423 groups, with 59 production services connected to it.

## Supporting federated data sharing for genomic health

Genomic technologies have advanced such that generating human genomic sequence data is no longer prohibited by cost and time. It is projected that by 2025, over 60 million patients will have their genome sequenced in a healthcare context (Birney *et al.*, 2017). Infrastructure for secure access to sensitive human data is not currently developed and there is often little opportunity for the reuse of data outside, or beyond, the core project. ELIXIR is driving the strategy toward sustainable and long-term infrastructure for sensitive human data in Europe (Saunders *et al.*, 2019). For example, the ELIXIR Federated Human Data Community develops solutions to overcome the access difficulties for data resources containing genomics and linked phenotypic and clinical data that are posed by general data protection regulations (GDPR) policies (Phillips, 2018). The ELIXIR Rare Diseases Community (https://elixir-europe.org/communities/rare-diseases), in partnership with RD-CONNECT (http://rd-connect.eu/), BBMRI-ERIC (http://bbmri-eric.eu/) and E-Rare (http://www.erare.eu/), is creating a federated infrastructure that will enable researchers to discover, access and analyze different rare disease repositories across Europe. This community is using Beacon (Fiume *et al.*, 2019), a genetic variation discovery tool initially developed by the Global Alliance for Genomics and Health (GA4GH), to share variant information from consented sensitive human genetic data stored in databases affiliated with ELIXIR Nodes and in the European Genome-phenome Archive (EGA).

GA4GH is an international, nonprofit alliance formed in 2013 to accelerate the potential of research and medicine to advance human health (https://www.ga4gh.org/about-us/). ELIXIR has been a formal contributor to the generation of GA4GH products (https://www.ga4gh.org/genomic-data-toolkit/) since the formation of the alliance. The ELIXIR Beacon project was one of the first GA4GH Driver Projects (Fiume *et al.*, 2019). In January 2017, to further develop and implement the Beacon technology across ELIXIR Nodes, the ELIXIR Beacon project adopted new ELIXIR AAI features such as tiered access and improved security, to minimize risk around individual privacy. The project has over 16 000 users accessing the Beacon API and to date more than 70 Beacons have been lit. In May 2019, a Strategic Partnership between ELIXIR and the GA4GH was announced (https://elixir-europe.org/news/elixir-and-ga4gh-expand-collaboration), building on the existing collaboration, and will facilitate the access to sensitive data across Europe, in order to help create virtual cohorts with tens of millions of participants. The coordinated development and deployment of the ELIXIR Beacon and ELIXIR AAI standards along with the suite of standards from the GA4GH Work Streams will help translate this vision into reality.

## ELIXIR addresses the problem of long-term sustainability for key bioinformatics resources

ELIXIR’s mission includes ensuring that all of Europe’s life science projects have access to long-term sustainable infrastructure for data management (Martin *et al.*, 2019). The five strategic objectives of the ELIXIR 2019–2023 Scientific Programme (https://elixir-europe.org/about-us/what-we-do/elixir-programme) include that ‘ELIXIR Core Data Resources will be the global standard for bioinformatics resource management and the foundation for an international funding and life cycle management strategy that secures the long-term sustainability of those resources’. Defined according to a protocol based on 23 indicators described in the article ‘Identifying ELIXIR Core Data Resources’ (Durinx *et al.*, 2017), the ELIXIR Core Data Resources (https://elixir-europe.org/platforms/data/core-data-resources) are a set of European data resources of fundamental importance to the wider life science community and the long-term preservation of biological data.

Having defined this set, ELIXIR has conducted an analysis to demonstrate scale of content and usage, open data and FAIR data leadership, impact, integration, interdependencies and precarious assured funding for these foundational data resources (Drysdale *et al.*, 2020). This is the first such demonstration, and, consequently, ELIXIR is participating in the Global Biodata Coalition (Anderson, 2017; Anderson *et al.*, 2017), an initiative involving an international group of funders [including Wellcome Trust (UK), NIH (US), AMED (JP) and A-STAR (SG)] and working toward long-term sustainability of Core Data Resources worldwide. Consideration has been given to potential alternative funding models (Gabella *et al.*, 2018) and it is clear that this challenge can only be addressed in international collaborations. Although long-term sustainability needs to extend beyond core data resources, this work has enabled focusing attention on the imperative to resolve life science bioinformatics resources, service and training sustainability, particularly as we enter a period of expansion of genomic health and diagnostic-related bioinformatics.

The long-term sustainability of the life science data ecosystem depends also on the adoption of standardized file formats, metadata, vocabularies and identifiers across data resources. Consequently, ELIXIR has established a portfolio of Recommended Interoperability Resources (https://elixir-europe.org/platforms/interoperability/rirs) to facilitate interoperability of life science data, to support the principles of FAIR data management, and to enable maximal reuse, and thereby value, of life science data, in the long term.

## Challenges and opportunities as ELIXIR matures

The first ELIXIR Scientific Programme 2014–2018 (https://elixir-europe.org/about-us/what-we-do/elixir-programme-2014-2018) was supported by ELIXIR Core funding and the Horizon 2020 ELIXIR-EXCELERATE grant (European Commission Research Infrastructures Programme of Horizon 2020, grant number 676559), enabling the evolution from a nascent to an established research infrastructure. As shown through the examples mentioned above, ELIXIR has laid the foundation for a sustainable life science ecosystem that underpins its 2019–2023 Scientific Programme (https://elixir-europe.org/about-us/what-we-do/elixir-programme). The 2019–2023 Programme transitions ELIXIR initiatives from the proof-of-concept stage to operating at scale.

This transformation of scale is evident in several dimensions. Whereas previously, Implementation Studies may have involved two or three Nodes with few participants, running for 12–18 months, ELIXIR now operates as a truly distributed European infrastructure in multiple contexts. For example, the *Federated Human Data* Implementation Study (https://elixir-europe.org/about-us/implementation-studies/federated-human-data) engages 26 collaborators in 13 Nodes organized into five work packages, and will run for 31 months. The plan includes a structured roadmap for ELIXIR Nodes to join the European Genome-phenome Archive (EGA) federated network by providing the necessary technical, logistical and training coordination across the network. This will enable population-scale genomic, phenotypic and biomolecular data to be accessible across international borders and provide a foundation for major initiatives such as the recent declaration by 21 European countries to transnationally provide access to at least 1 million human genomes from national programs by 2022 (Saunders *et al.*, 2019).

Connecting secure, federated clouds with open tool registries, packaging and workflow execution standards will enable analysis workflows to operate on data across national borders. Open repositories and support for good development practice will drive data reproducibility and provenance, which are of high importance in both research and clinical practices. The ELIXIR Implementation Study *Deploying Reproducible Containers and Workflows Across Cloud Environments* (https://elixir-europe.org/about-us/implementation-studies/containers-workflow-cloud), addresses the need for a stable infrastructure for unifying software container solutions within ELIXIR. This infrastructure will provide an access point for end-users to find, generate, store, monitor and even benchmark software container solutions. Such studies drive collaboration: the technical experts in ELIXIR’s Compute and Tools Platforms will work with developers and researchers in the Galaxy, Metabolomics and Proteomics Communities. In both of these examples, the scale of adoption and participation, as well as the ambition for generating reusable infrastructure across diverse communities is only possible because of the groundwork done over the 5 years.

After 5 years of operation, ELIXIR links 23 Nodes and builds on national initiatives to jointly develop and operate foundational services that enable data access, integration, analysis and training for the research community, transnationally. ELIXIR has been recognized as a Research Infrastructure of Global Interest by the G7 (https://ec.europa.eu/info/sites/info/files/research_and_innovation/gso_progress_report_2017.pdf) and brings together people, expertise and resources from more than 220 institutes. A key feature of ELIXIR’s strategy is to build long-term investments around services identified as strategically important, via community consultation. ELIXIR’s focus on connecting and adding value to the national Nodes via a jointly defined portfolio of shared services lays a foundation for long-term sustainable operations—grounded in national research infrastructure roadmaps. This contrasts with other initiatives such as the US-based BigData to Knowledge (BD2K) program (Bourne *et al.*, 2015; Dunn and Bourne, 2017) where a common infrastructure was anticipated; however, the time-limited, research-project-based funding period was followed by difficulty securing support for the necessary maintenance, after the conclusion of the initial BD2K program.

ELIXIR has embarked upon its second Scientific Programme (2019–2023), which lays out the scientific direction jointly agreed between ELIXIR Nodes. Significantly, ELIXIR has been awarded an EU H2020 3-year grant for data sharing and management, ELIXIR-CONVERGE (https://elixir-europe.org/about-us/how-funded/eu-projects/converge). This project will engage every Node across ELIXIR, and highlights ELIXIR’s vital role in driving good data management, reproducibility and reuse in a distributed and heterogeneous funding landscape.

In the biomedical domain, ELIXIR’s experience in operating large infrastructure consortia provides the basis to translate genomics from biomedical research into routine application in healthcare systems. ELIXIR Nodes are involved in several large European-funded genomic health projects such as European Joint Programme on Rare Diseases (https://www.ejprarediseases.org/) (EJP-RD) and Common Infrastructure for National Cohorts in Europe, Canada and Africa (https://www.cineca-project.eu/) (CINECA) and ensuring wider adoption of Node services and tools within the health system. ELIXIR is also coordinating the FAIRplus (https://fairplus-project.eu/) project, establishing FAIR data practices, increasing discovery, accessibility and reusability of data from selected projects funded by the EU’s Innovative Medicine Initiative, and internal data from seven pharmaceutical industry partners.

In plant and agriculture research a data federation spanning Europe’s largest plant phenotyping centers is now fully operational (Pommier *et al.*, 2019). This provides the foundation of a European federation of data repositories dedicated to plants, which will ultimately allow the exploration of distributed plant ‘omics datasets’, transnationally. ELIXIR’s participation in the Global Biodata Coalition signifies that the ‘well defined and transparent indicators’ (Anderson *et al.*, 2017) developed by the ELIXIR Data Platform to identify Core Data Resources and Deposition Databases are recognized by the wider international community, demonstrating ELIXIR’s role in driving Open Access as a foundational principle for publicly funded research.

ELIXIR is coordinating the EOSC-Life project (http://www.eosc-life.eu/), where the 13 ESFRI life sciences Infrastructures in Europe (https://www.esfri.eu/) join forces to create an open collaborative digital space for life science in the EOSC. The project aims to publish data from the life sciences Infrastructure facilities and centers as FAIR Data Resources in the cloud, link reusable Tools and Workflows to standardized compute services in national life science clouds, connect all users across Europe to a single login AAI system, and to develop joint data policies to preserve and deepen the trust given by research participants and patients volunteering their data and samples.

ELIXIR has well-established collaborations with a number of international initiatives such as GA4GH (see above) and the RDA (Research Data Alliance; https://www.rd-alliance.org/). In addition, it is establishing new collaboration strategies with national data infrastructures in key international countries such as the Australian Bioinformatics Commons Initiative (Schneider *et al.*, 2019) around tooling and compute infrastructure, so that both groups will benefit and increase their knowledge base. ELIXIR is also now extending engagement with the wider international community on initiatives such as H3Africa (https://h3africa.org/), whose vision is to support a network of pan-continental labs to study the complex interplay between environment and genetic factors, and investigate disease susceptibility and drug response across Africa.

In conclusion, ELIXIR is established as a sustainable infrastructure for managing and analyzing large and geographically distributed datasets based on global standards and shared components that add value to national investments. The commitment from ELIXIR’s member countries, as described in the ELIXIR 2019–2023 Programme, is to jointly provide services that enable European researchers and their collaborators to routinely access, analyze and reuse large, complex and geographically distributed datasets. Over the next 5 years, ELIXIR will deepen the existing partnerships and open up collaborations with new areas such as single-cell genomics, artificial intelligence and machine learning, and biodiversity research, to drive the vision to support life science research and its translation to society, the bioindustry, environment and medicine.
